# Volumetric Assessment and Graph Theoretical Analysis of Thalamic Nuclei in Essential Tremor

**DOI:** 10.1002/brb3.70346

**Published:** 2025-02-19

**Authors:** Maria Giovanna Bianco, Maria Eugenia Caligiuri, Camilla Calomino, Maria Celeste Bonacci, Valerio Aquila, Jolanda Buonocore, Antonio Augimeri, Alessia Sarica, Maria Grazia Vaccaro, Aldo Quattrone, Andrea Quattrone

**Affiliations:** ^1^ Neuroscience Research Center Magna Graecia University Catanzaro Italy; ^2^ Department of Medical and Surgical Sciences Institute of Neurology Magna Graecia University Catanzaro Italy; ^3^ Biotecnomed S.C.aR.L Catanzaro Italy

**Keywords:** essential tremor, graph analysis, thalamic nuclei

## Abstract

**Introduction:**

Essential tremor (ET) is a neurological disorder primarily characterized by upper limb action tremor. It is widely recognized that the thalamus is implicated in ET pathophysiology, playing a central role in treatment approaches. This study aimed to explore thalamic morphology, assessing macrostructural changes and intrinsic thalamic networks in ET patients.

**Methods:**

A total of 109 ET (41 with and 68 without resting tremor) and 81 healthy controls (HC) were enrolled in the study. An automatic probabilistic segmentation of thalamic nuclei was employed on T1‐weighted MRI images using FreeSurfer 7.4. Subsequently, volumetric data were extracted, and graph theoretical analysis was applied on the cortical–thalamic nuclei network, assessing global and local network properties.

**Results:**

No significant differences were observed in the volume of thalamic nuclei between ET patients and HC. ET patients exhibited significant alterations in the global thalamic network, suggesting a less efficient brain network in comparison with HC. ET patients also showed local alterations of thalamic network such as lower eccentricity and path length in the ventral nuclei and reduced efficiency in the pulvinar, indicating a less interconnected network. No significant differences were observed between ET patients with and without rest tremor.

**Conclusion:**

Our study demonstrates reduced global and local efficiency of brain networks in ET patients, suggesting impaired communication and interconnection between brain regions. These findings confirm the involvement of the ventral lateral and pulvinar nuclei as key regions in tremor pathophysiology in ET patients, supporting the targeting of these regions for therapeutic approaches.

## Graphical abstract

Graph theoretical analysis reveals reduced global and local network efficiency in thalamic nuclei of essential tremor patients, highlighting ventral lateral and pulvinar nuclei as key regions in tremor pathophysiology and potential therapeutic targets.

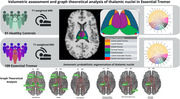

## 1 Introduction

Essential tremor (ET) is characterized by bilateral tremor in the upper limbs, at times associated with resting tremor or other subtle neurological signs (ET plus; Bianco et al. 2023). The exact interpretation of these additional symptoms remains a topic of ongoing discussion, and the underlying mechanism of ET remains incompletely understood (Bhatia et al. 2018). The thalamus holds a central role in modulating abnormal oscillatory tremor activity (Nicoletti et al. 2020) and it is a key target region in ET patients for tremor‐advanced treatments such as deep brain stimulation (DBS), MR‐guided focused ultrasound (MRgFUS), and radiofrequency thalamotomy (Agrawal et al. 2021; Barbagallo et al. 2018; Neudorfer et al. 2024; Madelein van der Stouwe et al. 2020).

To date, the thalamic ventral intermediate (VIM) nucleus is the most common target for these treatments, representing a viable treatment option for ET tremor symptoms refractory to oral medications (Agrawal et al. 2021). However, recent studies have shown that the stimulation of areas adjacent to the VIM, such as the ventral border, the ventrolateral or posterior (VL/VLp) thalamus, or the area beneath known as the posterior subthalamic area (PSA), can be equally or more effective (Agrawal et al. 2021).

The thalamus, traditionally considered as a relay station for sensory signals, is now recognized as a complex nuclear structure with diverse functions in sensory, cognitive, and motor processing. It comprises various nuclei, each with distinct connectivity and roles in emotional processing, reward evaluation, fear extinction, and motor control (Weeland et al. 2022). Recent studies have highlighted the thalamus involvement in these domains, expanding our understanding beyond its traditional perception. Neuroimaging techniques provided valuable insights into the pathophysiological mechanisms of tremor, revealing structural, neurotransmitter, and circuit alterations implicated in its manifestation (Weeland et al. 2022). Most neuroimaging studies in ET patients explored DTI/functional connectivity in the tremor network, confirming the pivotal role in the cerebello–thalamo–basal ganglia–cortical loop (Madelein van der Stouwe et al. 2020; Weeland et al. 2022; Caligiuri et al. 2022; Nigro et al. 2019). A recent study (Bagarinao et al. 2024) has proposed an alternative tremor network, with widespread functional connections of the pulvinar–cortical (visuomotor) and dorsomedial thalamus–cerebellar motor pathways. No studies, however, investigated in detail the macroscopic properties of the various thalamic subnuclei and their interconnection with cortical areas in ET patients.

The aim of this study was to investigate the cortical–thalamic nuclei network based on volumes using a graph theory to elucidate how the morphological properties of different thalamic nuclei are related to the cortex. Graph theory offers a powerful mathematical approach for analyzing topological properties of the brain networks (Bullmore and Sporns 2009). By modeling the brain as a graph, where nodes represent brain regions and edges represent the connections between them, it is possible to examine the structural aspects of neural networks. In the current study, we hypothesized that there were significant changes in structural interconnections between thalamic nuclei and the cortex in patients with ET exploiting graph theoretical network analysis.

## 2 Materials and Methods

### 2.1 Patients

The current study included 200 participants, 115 ET (45 with and 70 without resting tremor) and 85 control subjects. Patients and controls were recruited at the Institute of Neurology and the Neuroscience Research Center of the Magna Graecia University, Catanzaro, Italy between 2017 and 2024. All ET patients were diagnosed by the same movement disorder specialist using recent diagnostic criteria from the Movement Disorder Society task force (Bhatia et al. 2018). In addition, all patients underwent single photon emission computed tomography with 123I‐ioflupane (DaTscan; Quattrone et al. 2021), which was performed to exclude parkinsonian syndromes. Cognitive function was measured using the Mini‐Mental State Examinations (MMSE) for general cognitive impairment (Calomino et al. 2024), the Rey Auditory Verbal Learning Test immediate (RAVLT_I) and delayed recalls (RAVLT_D) for verbal learning and memory, the Controlled Oral Word Association Test (COWAT) for lexical stock, and the Digit Span Forwards (Digit Span F) and Backwards (Digit Span B) for attention and working memory (Bianco et al. 2023; Calomino et al. 2024), performed by the same physician. Control subjects were enrolled among partners of patients and had no history of neurological, psychiatric, or significant medical conditions. Exclusion criteria for ET patients encompassed potential dysmetabolic causes of tremor, abnormal DaTscan results, diffuse vascular lesions in the brain or lesions in the basal ganglia or brainstem on MRI, and current or past use of medications known to worsen or induce tremor, such as amiodarone, amphetamines, beta‐adrenergic agonists, antipsychotics, prednisone, lithium, and valproate. The study protocols and ethical considerations received approval from the institutional review board at Magna Graecia University in Catanzaro, Italy. All participants provided written informed consent before participating in the research.

### 2.2 MRI Acquisition and Image Processing

All subjects underwent brain MRI with the same 3‐T MR750 General Electric scanner with an eight‐channel head coil (Discovery MR‐750, GE, Milwaukee, WI, USA; Bianco et al. 2023). The acquisition protocol included three‐dimensional T1‐weighted volumetric spoiled gradient echo (GE), T2‐weighted fast spin echo, and T2‐weighted fluid attenuated inversion recovery sequences, as previously described (Bianco et al. 2023; Calomino et al. 2024).

Cortical and thalamic subnuclei segmentation was conducted using FreeSurfer 7.4.1 version (http://surfer.nmr.mgh.harvard.edu/) and the functional magnetic resonance imaging of the brain (FMRIB) Analysis Group Software Library (FSL 6.0.0; Oxford University, UK; Dale et al. 1999).

The cortical volumes (mm^3^) were segmented and extracted with the standard pipeline recon‐all (Dale et al. 1999). Briefly, nonbrain tissues were removed, and brains were aligned to Talairach space. Tissue types were classified into gray matter (GM), white matter (WM), and cerebrospinal fluid (CSF). The subcortical WM and deep GM volumetric structures were segmented and automated topology correction was applied. The above processing steps parcellated the cortex into 68 gyral‐based regions of interest (ROIs) according to the Desikan–Killiany atlas. Finally, each ROI was grouped into anatomical lobes, that is, frontal, parietal, temporal, occipital, and cingulate lobes.

**FIGURE 1:**
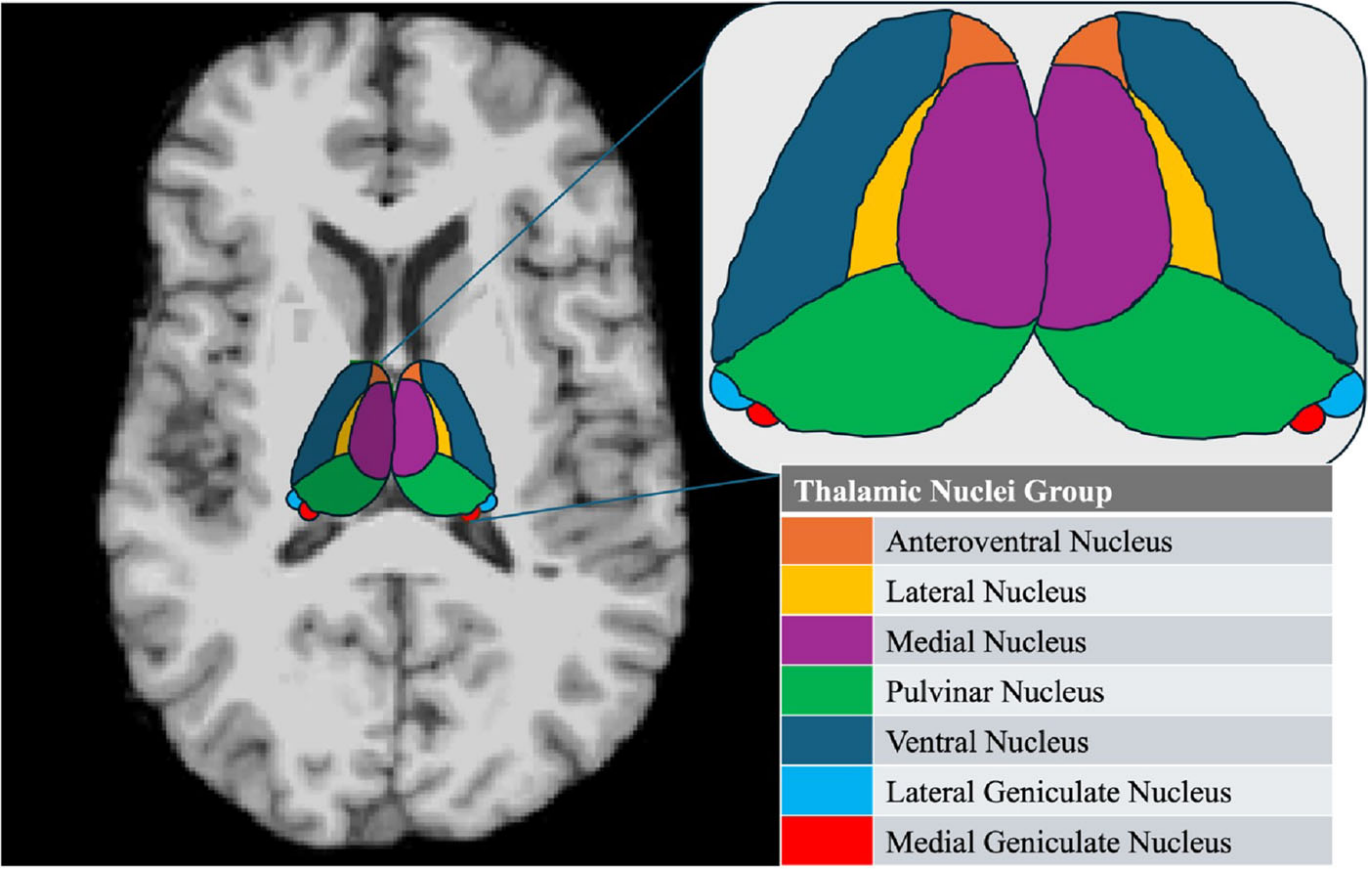
The figure shows how the thalamic nuclei were grouped. These groups included the anteroventral nuclei, the lateral nuclei (lateral dorsal nucleus and lateral posterior nucleus), the medial nuclei (central medial nucleus, paracentral nucleus, lateral mediodorsal nucleus [parvocellular], medial mediodorsal nucleus, parafascicular nucleus, centromedian nucleus, central lateral nucleus, reuniens nucleus, and paratenial nucleus), the pulvinar nuclei (anterior pulvinar nucleus, inferior pulvinar nucleus, lateral pulvinar nucleus, and medial pulvinar nucleus), the ventral nuclei (ventral anterior nucleus, ventromedial nucleus, ventral lateral anterior nucleus, ventral lateral posterior nucleus, and ventral posterolateral nucleus), and the lateral and medial geniculate nuclei.

Subsequently, thalamic nuclei were segmented applying Bayesian probabilistic atlas, derived from a combination of histological tissue data and structural MRI data (Iglesias et al. 2018) as described in the ENIGMA pipeline (Van den Heuvel et al. 2022; Boelens Keun et al. 2021; chrisvriend/ENIGMA_subthal). Briefly, FreeSurfer's recon‐all function generated thalamic segmentation, which was then overlaid with the probabilistic atlas by Iglesias et al. (2018), resulting in 25 thalamic nuclei in each hemisphere. Due to the small size of certain nuclei, they were grouped into larger nuclear groups based on their anatomical and functional characteristics: the anteroventral, ventral, medial, lateral, pulvinar, and lateral and medial geniculate nuclei, as previously described (Boelens Keun et al. 2021; Calomino et al. 2024) and showed in Figure 1. The quality control of the thalamic nuclei segmentation was carried out into the following steps. First, we assessed statistical outliers for each nucleus (below Q1 − 1.5 × IQR or above Q3 + 1.5 × IQR). Second, we examined the differences in voxels between the total thalamus volume segmented by FreeSurfer's recon‐all function and that segmented based on the probabilistic atlas (Iglesias et al. 2018; Van den Heuvel et al. 2022; Boelens Keun et al. 2021). In addition, subjects that segmentations showed a large overlap between thalamic GM with WM and CSF were flagged and visually inspected as well (Boelens Keun et al. 2021). Radiological assessment was conducted by the same two trained raters for all patients. Poor segmentations were excluded from the analysis, the final cohort consisted of 190 patients, 109 ET (41 with and 68 without resting tremor) and 81 control subjects.

### 2.3 Network Construction and Analysis

The intrinsic graph network between 10 cortical brain regions (frontal, parietal, temporal, occipital, and cingulate regions of both hemispheres) and the 12 thalamic nuclei was constructed using BRAPH (Brain Analysis using Graph theory; http://braph.org) software (Mijalkov et al. 2017). Before network construction, cortical volume and subcortical thalamic nuclei were adjusted for age, sex, and intracranial volume (ICV) using linear regression to remove their potential confounding effects.

Within this framework, nodes were established to each volume, and edges were defined as Pearson correlations, with negative correlations set to zero. A weighted, undirected connectivity matrix from nodes and edges was built for each group. The edges in the weighted undirected graphs indicated the strength of the connection and were symmetric and undirected (i.e., if node (i) is connected to node (j), then node (j) is also connected to node (i)).

To determine the differences between groups in the brain network, we calculated the following global and local properties. We extracted the global network measures from the connectivity matrix, such as average degree, average strength, radius, diameter, eccentricity, characteristic path length, global efficiency, clustering, transitivity, modularity, assortativity, and small‐worldness index. As local properties used to assess whether a node is a brain hub, we used betweenness centrality, closeness centrality, clustering nodes, degree, eccentricity, local efficiency nodes, participation, path length, strength, triangles, and within module degree *z*‐score (Mijalkov et al. 2017).

Significant between‐group differences on graph properties were investigated using nonparametric permutation tests with 1000 replications, as described in previous studies (Mijalkov et al. 2017; Benjamini and Hochberg 1995; Lenka and Jankovic 2021). In details, the subjects from both groups were first randomly reassigned. The differences in graph measures between these new randomized groups were then calculated. This permutation process was repeated 1000 times to generate a distribution of between‐group differences. The *p* values were determined by calculating the fraction of the difference distribution values that exceed the observed difference between the actual groups (Mijalkov et al. 2017). In addition, a false discovery rate (FDR) correction using the Benjamini–Hochberg procedure (*q* < 0.05) was applied to control for multiple comparisons (Benjamini and Hochberg 1995).

### 2.4 Statistical Analysis

All statistical analyses were performed in R (version 4.1.2; R Foundation for Statistical Computing, Vienna, Austria). Shapiro–Wilk test was employed to test normality of demographic and clinical data. Fisher's exact test was used to assess differences in sex distribution. Age at examination and education were compared with ANOVA or Kruskal–Wallis test for nonparametric variables. Cognitive tests were compared among groups with analysis of covariance (ANCOVA) with education and age as covariate. ANCOVA with sex, age, and estimated ICV as covariates was used to compare volumetric MRI data between groups. Correlation analysis (Spearman) between imaging data and age or disease duration were investigated. In all statistical analyses a *p* < 0.05 was considered as significant after FDR correction.

## 3 Results

Demographic, clinical and MRI data of patients and controls are summarized in Table 1. No differences were found in sex and education among groups. ET patients were slightly older and had a MMSE score significantly lower than HC (Table 1). No demographic and clinical differences were found between pure ET patients (*n* = 68) and ET patients with rest tremor (*n* = 41), as shown in Table S1. ET patients showed a slight volume increase of the whole right thalamus and of bilateral medial nuclei and pulvinar nuclei in comparison with HC (Table 2), not surviving FDR correction. No significant differences in cortical volumes were found among groups (Table 2). No significant differences in MRI volumes were found between ET and ET plus, which were thus considered as a whole group.

**TABLE 1:** Demographic and clinical data of patients with essential tremor and healthy controls.

Data	ET (*N* = 109)	HC (*N* = 81)	*p* value
Sex (M/F)	51/58	45/36	0.244^a^
Age at examination, years^b^	64.7 ± 11.0	63.3 ± 8.20	0.025^c^
Disease onset, years^b^	51.3 ± 16.9	—	—
Disease duration, years^b^	14.4 ± 14.6	—	—
Education, years^b^	9.58 ± 4.97	10.7 ± 4.21	0.222^c^
Fahn–Tolosa–Marín Tremor Rating Scale^b^	20.2 ± 17	—	—
MMSE^b^	26.0 ± 3.11	28.1 ± 1.66	0.001^d^
COWAT^b^	23.9 ± 6.53	29.0 ± 10.8	0.084^d^
RAVLT_RI^b^	36.6 ± 10.4	37.1 ± 7.68	0.858^d^
RAVLT_RD^b^	6.66 ± 2.75	6.73 ± 2.34	0.949^d^
DIGIT_SPAN_F^b^	4.95 ± 0.84	5.31 ± 0.93	0.793^d^
DIGIT_SPAN_B^b^	3.21 ± 0.85	3.38 ± 0.80	0.760^d^

*Note*: Cognitive tests were available in a cohort subgroup as follows: MMSE, 54 ET, and 45 HC; COWAT, 22 ET, and 26 HC; RAVLT_RI, 40 ET, and 26 HC; RAVLT_RD, 39 ET, and 26 HC; DIGIT_SPAN_F,34 ET and 26 HC; DIGIT_SPAN_B,19 ET and 26 HC.

Abbreviations: ET, essential tremor; HC, healthy controls; MMSE, Mini‐Mental State Examination.

^a^ Fisher's exact test.

^b^ Data are expressed as mean ± standard deviation.

^c^ ANOVA or Kruskal–Wallis test where appropriate.

^d^ ANCOVA with age and education as covariates.

**TABLE 2:** Volumetric data of thalamus, thalamic nuclei, and cortical lobes in ET patients and healthy controls.

Data	ET (*N* = 109)	HC (*N* = 81)	*p* value
Left thalamus^a^	6229 ± 759	6109 ± 742	0.08^b^
Right thalamus^a^	6097 ± 722	5933 ± 695	0.02^b^
Left ventral^a^	2571 ± 358	2545 ± 355	0.255^b^
Left anteroventral^a^	108 ± 23	106 ± 16	0.272^b^
Left medial^a^	1381 ± 173	1345 ± 157	0.022^b^
Left lateral^a^	116 ± 27	116 ± 22	0.800^b^
Left pulvinar^a^	1666 ± 235	1609 ± 230	0.023^b^
Left LGN^a^	223 ± 42	227 ± 43	0.505^b^
Left MGN^a^	109 ± 18	106 ± 20	0.323^b^
Right ventral^a^	2485 ± 343	2431 ± 340	0.138^b^
Right anteroventral^a^	116 ± 23	114 ± 16	0.520^b^
Right medial^a^	1364 ± 170	1324 ± 139	0.009^b^
Right lateral^a^	110 ± 24	109 ± 22	0.827^b^
Right pulvinar^a^	1647 ± 231	1581 ± 215	0.009^b^
Right LGN^a^	210 ± 36	212 ± 35	0.974^b^
Right MGN^a^	114 ± 18	112 ± 20	0.485^b^
Left frontal^a^	55,288 ± 5561	55,298 ± 6209	0.943
Right frontal^a^	56,053 ± 5862	55,956 ± 6153	0.917
Left parietal^a^	47,449 ± 5102	47,103 ± 5742	0.462
Right parietal^a^	48,919 ± 4863	48,870 ± 5947	0.865
Left temporal^a^	48,159 ± 4940	47,991 ± 5740	0.836
Right temporal^a^	46,923 ± 4594	46,680 ± 5402	0.680
Left occipital^a^	20,261 ± 2628	20,127 ± 2841	0.726
Right occipital^a^	21,085 ± 2627	20,864 ± 3000	0.554
Left cingulate^a^	8717 ± 1301	8572 ± 1274	0.341
Right cingulate^a^	8196 ± 1118	8165 ± 1317	0.838

*Note*: No *p* value survived FDR correction.

Abbreviations: ET, essential tremor; HC, healthy controls.

^a^ Data are expressed as mean ± standard deviation.

^b^ ANCOVA with sex, age, and estimated intracranial volume as covariate.

**FIGURE 2:**
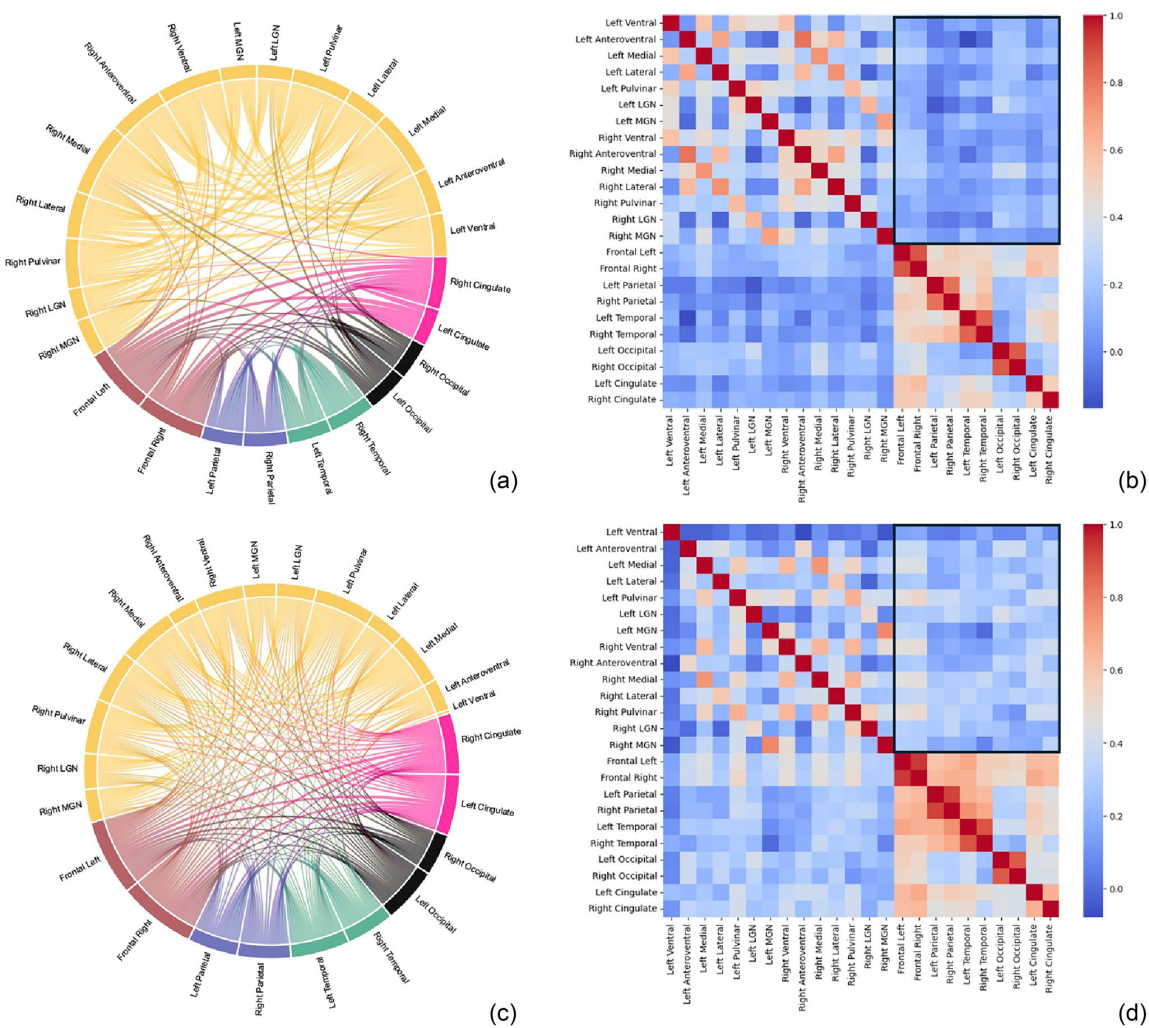
Chord diagram and adjacency matrices of undirected graphs network in essential tremor (a, b) and healthy controls (c, d). The figures show the partial correlation with age, sex, and total intracranial volume as covariates, from each ROI. Chord diagram shows thalamic nuclei group (yellow) and cortical volumes of frontal (red), parietal (blue), temporal (green), occipital (black), and cingulate (pink) lobes. A lower number of connections among nodes is visible in the ET‐derived chord diagram (a) compared to HC (c). For completeness, the corresponding adjacency matrices are shown on the right side of the figure (b, d), and the correlation strength is shown using a color bar. Several darker blue squares are observed in the ET‐derived matrix, reflecting lower correlations among cortical structures and thalamic nuclei volumes in this patient group.

The partial correlation matrices between ROIs are shown in Figure 2, with the chord diagrams (a, c) and adjacency matrices (b, d). Both groups demonstrated strong correlations between bilaterally homologous regions. Notably, the ET group had visibly lower correlations among cortical structures and thalamic nuclei in contrast to healthy subjects, reflecting weaker connectivity and less intense interactions between network nodes.

**FIGURE 3:**
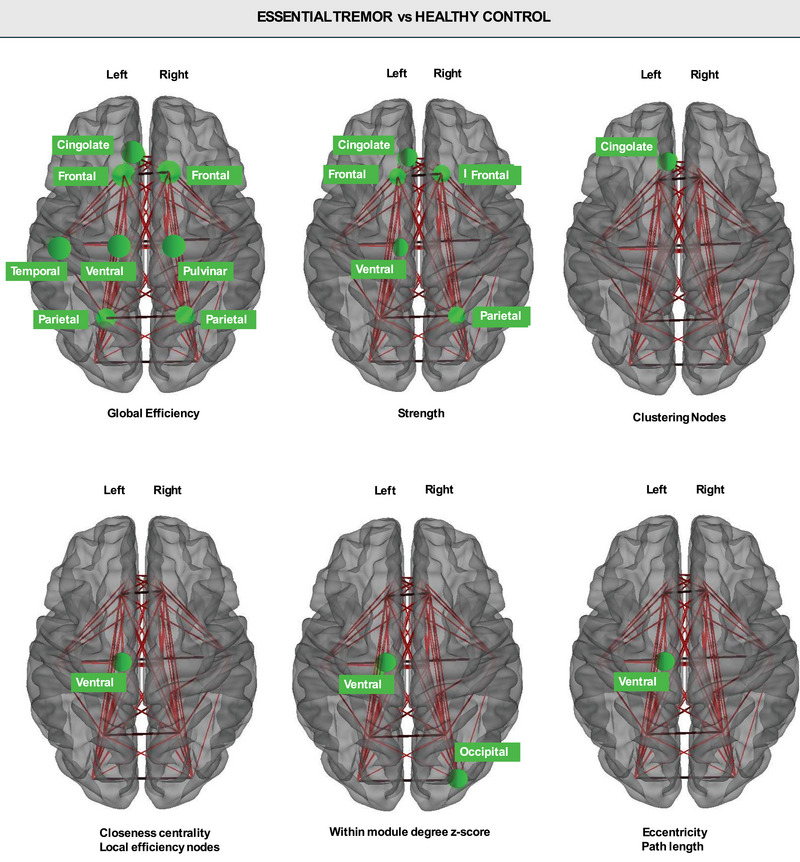
Brain regions with significant differences (*p* < 0.05 FDR corrected) in nodal properties between groups. This figure shows axial brain views.

In the global network analysis, ET patients showed a reduced average strength, clustering, transitivity, global efficiency, and small‐worldness index and increased modularity, eccentricity, and characteristic path length compared with HC (Tables 3 and S2). By investigating the network local properties, significant differences for nodal measures were found in the right pulvinar and the left ventral thalamic nucleus (Tables 4 and S2; Figure 3). The right pulvinar showed a reduced global efficiency in ET patients; the ventral region exhibited a series of nodal properties indicative of a lower path length and eccentricity of network and higher closeness centrality, global and local efficiency nodes, strength, and within module degree in patients with ET than in HC. Regarding cortical ROIs, the temporal, frontal, parietal, and cingulate lobes showed lower global efficiency, strength, and clustering nodes in ET patients compared to controls. Moreover, the occipital cortex node was less central within the network in ET compared to controls.

**TABLE 3:** Differences in global measures between essential tremor patients and healthy controls.

Global graph measure	ET	HC	CI lower	CI upper	Difference	*p* value	Comparison
Average strength	5.22	7.50	−1.67	1.87	2.28	0.034	ET < HC
Global efficiency	0.27	0.35	−0.05	0.07	0.08	0.048	ET < HC
Clustering	0.19	0.30	−0.08	0.09	0.11	0.035	ET < HC
Transitivity	0.29	0.46	−0.12	0.12	0.17	0.021	ET < HC
Small‐worldness	0.79	0.91	−0.16	0.19	0.12	0.001	ET < HC
Modularity	0.25	0.10	−0.10	0.11	−0.15	0.020	ET > HC
Eccentricity	7.25	6.94	−12.94	9.47	−0.31	0.001	ET > HC
Characteristic path length	4.66	3.43	−1.65	1.41	−1.23	0.001	ET > HC

*Note*: Differences between patients with essential tremor and healthy controls. CI lower and upper are the 95% confidence interval limits of the difference between the groups.

Abbreviations: ET, essential tremor; HC, healthy controls.

**TABLE 4:** Brain regions contributing to group differences in the nodal analysis.

Brain region	Local graph measure	ET	HC	CI lower	CI upper	Difference	*p* value	Comparison
Left ventral	Closeness centrality	0.22	0.15	−0.07	0.08	−0.07	0.024	ET > HC
Left ventral	Eccentricity	7.51	8.90	−13.91	10.93	1.39	0.024	ET < HC
Left ventral	Global efficiency nodes	0.27	0.15	−0.07	0.09	−0.12	0.024	ET > HC
Left ventral	Local efficiency nodes	0.36	0.18	−0.11	0.14	−0.18	0.024	ET > HC
Left ventral	Path length	4.64	6.83	−15.16	10.87	2.19	0.024	ET < HC
Left ventral	Strength	4.83	1.65	−1.38	1.70	−3.18	0.024	ET > HC
Left ventral	Within module degree *z*‐score	0.95	−2.55	−0.48	0.75	−3.50	0.024	ET > HC
Right pulvinar	Global efficiency nodes	0.26	0.39	−0.07	0.10	0.13	0.045	ET < HC
Left cingulate	Clustering nodes	0.15	0.34	−0.09	0.10	0.19	0.048	ET < HC
Left cingulate	Global efficiency nodes	0.25	0.40	−0.07	0.09	0.15	0.024	ET < HC
Left cingulate	Strength	4.39	9.06	−2.68	2.67	4.67	0.036	ET < HC
Left frontal	Global efficiency nodes	0.32	0.47	−0.08	0.10	0.15	0.024	ET < HC
Left frontal	Strength	7.00	10.82	−2.37	2.47	3.83	0.048	ET < HC
Right frontal	Global efficiency nodes	0.33	0.48	−0.08	0.10	0.15	0.024	ET < HC
Right frontal	Strength	7.04	10.99	−2.37	2.56	3.95	0.040	ET < HC
Right occipital	Within module degree *z*‐score	−1.47	0.97	−1.42	1.44	2.44	0.048	ET < HC
Right parietal	Global efficiency nodes	0.25	0.38	−0.07	0.08	0.13	0.024	ET < HC
Right parietal	Strength	4.13	8.13	−2.58	2.93	4.00	0.043	ET < HC
Left parietal	Global efficiency nodes	0.27	0.40	−0.06	0.08	0.13	0.024	ET < HC
Left temporal	Global efficiency nodes	0.27	0.41	−0.08	0.09	0.13	0.030	ET < HC

*Note*: The brain regions and metrics from nodal analyses that were significantly different between patients with essential tremor and healthy controls are shown (*p* < 0.05 FDR corrected). CI lower and upper are the 95% confidence interval limits of the difference between the groups.

Abbreviations: ET, essential tremor; HC, healthy controls.

No differences were found in global and local properties between ET with and without rest tremor.

## 4 Discussion

The current study examines graph analysis of thalamic nuclei in subjects with ET, revealing significant differences in both global and local properties within the cortical–thalamic nuclei network in comparison with healthy control subjects. These data suggest a widespread reorganization of the brain's structural network among thalamic nuclei and cortical regions in ET, indicating that the brain network of ET patients was overall less efficient and more compartmentalized, with some brain regions becoming more isolated and less interconnected.

The role of the thalamus in the pathogenesis of tremor is well‐recognized (Lenka and Jankovic 2021). Multiple studies have demonstrated increased functional connectivity within the cerebellum–thalamus–cortical network in patients with tremor, suggesting its involvement in the generation and modulation of abnormal oscillations (Miskin and Carvalho 2018). Furthermore, the effectiveness of thalamic interventions, such as thalamotomy and DBS of the VIM nucleus, in improving tremor symptoms in ET patients provides further evidence for the active involvement of a thalamic neuronal subpopulation in tremor generation (Nicoletti et al. 2020). Recent studies (Miskin and Carvalho 2018; Hwang et al. 2017; Kawabata et al. 2021) have found that thalamic nuclei exhibit network properties capable of integrating multimodal information from different cortical functional areas, making the thalamus a critical hub for integrating and processing information between cortical regions (Hwang et al. 2017).

In this study, graph‐based network analysis revealed significant alterations in both global and local properties in patients with ET compared to healthy controls (HC). We found that patients with ET showed a reduction in several key global network measures, including average strength, clustering, transitivity, global efficiency, and the small‐worldness index, leading to a diminished ability to integrate information across different regions (global efficiency). In addition, the decreases in clustering and transitivity found in ET indicated that the network regions were poorly connected to neighboring areas, suggesting a segregation behavior. This disruption in the small‐world network organization could be a key factor in the pathophysiology of ET, and is in line with a previous study in a small ET group (Jang et al. 2016; Benito‐León et al. 2019). Interestingly, a study conducted in ET patients who underwent thalamotomy with focused ultrasound revealed that the treatment may induce transient changes in the global network circuit, further corroborating this hypothesis (Jang et al. 2016).

In addition, we found that ET patients presented an increased modularity, eccentricity, and characteristic path length. These changes indicated the brain networks of ET patients were more compartmentalized, with distinct modules that might be less interconnected. The longer characteristic path length and higher eccentricity suggested that the information flow across the network was less efficient, with some brain regions becoming more isolated in ET patients. Overall, the brain network organization appeared more segregated, where different brain regions operated more independently, suggesting that these network disruptions may underlie symptoms associated with ET.

Considering local network measures, we found significant differences in the thalamic left ventral and right pulvinar nuclei between ET patients and HC. The VIM nucleus, contained in the ventral nucleus, is the traditional target for selective thermal ablation using transcranial MR‐guided focused ultrasound (MRgFUS) thalamotomy or DBS, as these interventions provide immediate and effective tremor reduction. Several neuroimaging studies have localized in the ventral nucleus and in the motor cortex, the key brain regions involved in the motor component of ET (Revuelta et al. 2019; Raethjen and Deuschl 2012; Hallett 2014; Sharifi et al. 2014; Bolton et al. 2022). Although the exact pathophysiological mechanisms remain debated, ET is believed to result from a dysregulation of these interacting areas (Sharifi et al. 2014). In this regard, graph theory gives insights into how information flows in a complex system, such as the thalamic ventral nucleus and the cortical areas. In the current study, the left ventral nucleus including VIM had a higher global and local efficiency, closeness centrality, and strength in processing and integrating information in ET patients compared with HC. Moreover, ET showed a lower eccentricity and path length than controls, suggesting a more compact and efficiently wired network. Overall, these results suggest that a “hyperefficient” network including these thalamic structures and cortical regions may be a key alteration in ET. This finding is in line with a previous study showing that ET patients exhibited higher VIM–cortical connectivity in diffusional kurtosis imaging, which was associated with greater tremor severity (Revuelta et al. 2019), and in line with the clinical benefit usually observed after therapeutic approaches targeting this thalamic region.

Notably, in our study, we also found altered network properties, particularly a reduction in global efficiency, in the right pulvinar. In detail, we found a lower structural connectivity pattern of pulvinar with cortical structures in ET patients, suggesting decreased efficiency in message transmission across these regions. Higher order nuclei like the pulvinar receive inputs from cortical layer 5 and are involved in cortico–thalamo–cortical circuits, which are implicated in visual attention and other cognitive processes (Fischer and Whitney 2012; Saalmann et al. 2012; Sherman 2016; Mo and Sherman 2019). In detail, the pulvinar relays information between cortical areas, such as from primary to secondary visual regions (V1 to V2), through a transthalamic pathway critical for sensorimotor processing. Structural connections between the pulvinar and visual cortex have been demonstrated in humans and nonhuman primates (Saalmann et al. 2012; Mo and Sherman 2019; Fischer and Whitney 2012). In addition, projections from the frontal eye fields and intraparietal sulcus, via the superior colliculus, form loops with the pulvinar that integrate visual and dorsal attention networks to coordinate attentional functions (Greene et al. 2020). In addition to the lower structural connectivity pattern of pulvinar with cortical structures, we also observed that the occipital lobe, which is the primary region of the brain responsible for processing visual information, exhibited a lower centrality and fewer connections to other nodes in the network of ET patients. Overall, these findings may suggest the involvement of visual attention and visuospatial circuits in ET, with the pulvinar dysfunction possibly contributing to the nonmotor symptoms of ET, such as attention deficits and visuospatial impairments, by disrupting the integration of visual and attentional networks.

Our study has several strengths. First, we included a quite large group of ET patients, including both patients with classical ET and patients with ET plus, which are often underrepresented in research on ET syndrome. Second, all MRI data were acquired with the same protocol and underwent rigorous quality control procedures, adhering strictly to ENIGMA guidelines and were acquired through fully automated and validated methodologies.

Limitations should be considered when interpreting our results. First, the absence of postmortem pathological examinations in ET patients leaves open the possibility of misdiagnosis in a few cases. However, it's important to note that all patients were diagnosed according to recent international diagnostic criteria, and all patients underwent DaTscan imaging, which was normal, ruling out parkinsonian syndromes. Second, the control group was relatively smaller than the ET patient group, and there was a slight age difference between ET patients and healthy subjects. We addressed this limitation by including age as covariate in all analyses to mitigate potential biases. Future studies on larger ET and HC cohorts are warranted to confirm our findings. A further limitation is the absence of functional imaging or other modalities, such as magnetoencephalography or surface electromyography (Laganà et al. 2024; Aracri et al. 2024), which could provide additional insights into real‐time brain connectivity and dynamic network alterations in ET patients. On the other hand, structural imaging is more accessible and less sensitive to patient motion, enhancing its clinical applicability. Future studies integrating both structural and functional imaging approaches may yield a more comprehensive understanding of the pathophysiology underlying ET and its subtypes.

## 5 Conclusion

Our study revealed significant alterations in the structural connectivity patterns of thalamic nuclei in patients with ET. The analysis of global and local brain connectivity demonstrated inefficiency in information flow within the cortical–thalamic circuits; in addition, the VIM nucleus seems to be a hyperconnected thalamic nucleus implicated in tremor, whereas the pulvinar was a hypoconnected region, suggesting the involvement of visual attention and visuospatial circuits in ET.

Moreover, differences in graph properties between ventral and pulvinar nuclei pave the way for targeted therapeutic strategies to restore network efficiency and improve clinical outcomes in ET patients. Our results confirm the central role of thalamus in ET, support the targeting of VIM for therapeutic approaches, and provide new insights into the brain network involved in ET.

Future studies may combine structural and functional MRI data to comprehensively explore the dysfunction of networks involving thalamic nuclei in ET. Moreover, our results pave the way for longitudinal studies investigating changes in thalamic nuclei structure and connectivity over the disease course and to evaluate the possible effect of therapeutic strategies on network dynamics. Current and future research may ultimately clarify the potential translational value of these imaging data in advancing personalized approaches to tremor management.

## Author Contributions

**Maria Giovanna** **Bianco**: conceptualization, investigation, methodology, validation, visualization, writing–review and editing, writing–original draft, software, data curation, formal analysis. **Maria Eugenia Caligiuri**: data curation, software, writing–original draft, writing–review and editing. **Camilla Calomino**: software, data curation, methodology, validation, visualization, writing–review and editing. **Maria Celeste Bonacci**: methodology, validation, visualization, software, data curation, writing–review and editing. **Valerio Aquila**: writing–review and editing, data curation. **Jolanda Buonocore**: data curation, writing–review and editing. **Antonio Augimeri**: software, methodology, writing–review and editing, resources, data curation. **Alessia Sarica**: data curation, writing–review and editing, visualization. **Maria Grazia Vaccaro**: data curation, writing–review and editing. **Aldo Quattrone**: writing–review and editing, methodology, conceptualization, writing–original draft, software, validation, investigation, data curation, funding acquisition, supervision, formal analysis, visualization, resources, project administration. **Andrea Quattrone**: conceptualization, investigation, funding acquisition, writing–original draft, writing–review and editing, methodology, data curation, supervision, resources, visualization, project administration.

## Ethics Statement

All patients in our study gave their informed consent prior to their inclusion in the study. Approval of our study was obtained from the ethics committee of Magna Graecia University review board, Catanzaro, Italy. The procedures used in this study adhere to the tenets of the Declaration of Helsinki.

## Consent

Informed consent was obtained from all individual participants included in the study. All patients also signed informed consent regarding publishing their data.

## Conflicts of Interest

The authors declare no conflicts of interest.

### Peer Review

The peer review history for this article is available at https://publons.com/publon/10.1002/brb3.70346

## Supporting information



Supporting Information

## Data Availability

The data that support the findings of this study are available on request from the corresponding author. The data are not publicly available due to privacy or ethical restrictions.
