# Supplementary material for: Volumetric Assessment and Graph Theoretical Analysis of Thalamic Nuclei in Essential Tremor
Source: Brain Behav. 2025 Feb 19;15(2):e70346. doi: 10.1002/brb3.70346 (PMC11839756; doi:10.1002/brb3.70346)
Supplement: Supplementary file 1 — Supporting Information [file BRB3-15-e70346-s001.docx]

**Supplementary Materials**

**Supplementary Table 1.** Demographic and clinical data of patients with essential tremor and essential tremor plus

| **Data** | **ET**  **(N=68)** | **ET plus**  **(N=41)** | **p-value** |
| --- | --- | --- | --- |
| Sex, (M/F) | 30/38 | 21/20 | 0.553^a^ |
| Age at examination, ys^b^ | 64.6 ± 10.4 | 64.8 ± 12.1 | 0.632^c^ |
| Disease onset, ys^b^ | 52.5 ± 17.2 | 49.7 ± 16.6 | 0.366^c^ |
| Disease duration, ys^b^ | 12.4 ± 14.0 | 16.3 ± 14.9 | 0.052^c^ |
| Education, ys^b^ | 10.2 ± 5.16 | 7.73 ± 3.98 | 0.167^c^ |
| Fahn-Tolosa-Marín tremor rating scale^b^ | 18.6 ± 112.2 | 23.1 ± 14 | 0.254^d^ |
| MMSE^b^ | 26.0 ± 3.52 | 26.1 ± 2.47 | 0.114^e^ |
| COWAT^b^ | 23.2 ± 6.24 | 24.8 ± 7.08 | 0.482 ^e^ |
| RAVLT_RI^b^ | 37.6 ± 10.7 | 33.7 ± 9.45 | 0.756 ^e^ |
| RAVLT_RD^b^ | 6.74 ± 2.87 | 6.40 ± 2.48 | 0.856 ^e^ |
| DIGIT_SPAN_F^b^ | 4.89 ± 0.84 | 5.10 ± 0.86 | 0.517 ^e^ |
| DIGIT_SPAN_B^b^ | 3.44 ± 0.88 | 3.00 ± 0.82 | 0.583 ^e^ |

Abbreviations: ET= essential tremor; HC= healthy controls; MMSE = Mini Mental State Examination; ys = years.

^a^Fishers exact test; ^b^Data are expressed as mean ± standard deviation; ^c^ ANOVA or Kruskal-Wallis test where appropriate,^d^ Independent sample t-test, ^e^ANCOVA with age and education as covariates.

Cognitive tests were performed on: MMSE, 32 ET and 22 ET plus; COWAT, 12 ET and 10 ET plus; RAVLT_RI, 30 ET and 10 HC; RAVLT_RD, 29 ET and 10 HC; DIGIT_SPAN_F,24 ET and 10 HC; DIGIT_SPAN_B,9 ET and 10 HC.

**Supplementary Table 2.** Detailed explanation of global and nodal properties in a network

| **GLOBAL MEASURE** | **DESCRIPTION** |
| --- | --- |
| average strength | The strength is the sum of the weights of all edges connected to a node. It represents the number of connections between nodes and the weight of these connections in the cortical-nuclei network. This is the average strength of all nodes. |
| global efficiency | This refers to how efficiently information can be transmitted through the brain network. It measures the efficiency of the average path through the brain. |
| clustering | The clustering coefficient measures the tendency of nodes to form clusters or groups of highly interconnected nodes. |
| transitivity | Transitivity measures the proportion of triangles (three interconnected nodes) relative to the total number of triads (three nodes with at least one connection between them) in the network. |
| small-worldness | Small-worldness quantifies how the network is optimized both for local efficiency (high clustering) and for global efficiency (short path lengths). |
| modularity | Modularity measures the ability of the network to be divided into distinct modules or communities, with high connection density within modules and low connection density between modules. Its calculation requires a previously determined community structure. |
| eccentricity | The eccentricity of a node is the maximal distance between that node and any other node in the network. |
| char. path length | This is the average distance from a node to all other nodes in the network. |
| **NODAL MEASURE** |  |
| closeness centrality | Closeness centrality provides a measure of how close a node is to all other nodes. It is defined as the inverse of the sum of the shortest distances from a node to all other nodes. The shorter the total distance in the network, the higher the closeness centrality. In other words, it represents how quickly information can reach other nodes from a given starting node. |
| eccentricity | The eccentricity of a node is the maximum distance between that node and any other node in the network. A node with high eccentricity is more "isolated," as there are some nodes that are far away. Low eccentricity indicates that the node is relatively close to all other nodes. |
| global efficiency nodes | Global efficiency measures the inverse of the average shortest path lengths between all nodes in the network. When applied to individual nodes, it measures how efficiently a node can exchange information with all other nodes. A node with high global efficiency is highly efficient in transmitting information across the network, indicating an important role in network communication. |
| local efficiency nodes | Local efficiency measures the efficiency of the local subgraphs, or subnetworks, formed by a node's neighbors. A node with high local efficiency is part of a highly connected subnetwork, implying strong local cohesion and communication capability within the node's neighborhood. |
| path length | Path length measures the average distance between a node and all other nodes in the network, calculated as the average of the shortest path distances. |
| strength | A node with high strength has many strong connections, suggesting that it is a key node in the network regarding communication and information integration. |
| Within-module z-score | This is a measure of a node's centrality within its module. A module, or community, is a group of nodes that are more densely connected to each other than to nodes in other modules. |
